# Gene Expression Microarray Data Identify Hub Genes Involved in Osteoarthritis

**DOI:** 10.3389/fgene.2022.870590

**Published:** 2022-06-06

**Authors:** Jian Zhou, Dazhi Zou, Rongjun Wan, Jie Liu, Qiong Zhou, Zhen Zhou, Wanchun Wang, Cheng Tao, Tang Liu

**Affiliations:** ^1^ Department of Orthopedics, The Second Xiangya Hospital of Central South University, Changsha, China; ^2^ Department of Spine Surgery, Longhui People’s Hospital, Shaoyang, China; ^3^ Branch of National Clinical Research Center for Respiratory Disease, Department of Respiratory Medicine, National Key Clinical Specialty, Xiangya Hospital, Central South University, Changsha, China; ^4^ National Clinical Research Center for Geriatric Disorders, Xiangya Hospital, Changsha, China; ^5^ Department of Cardiology, The Fourth Hospital of Changsha, Changsha, China; ^6^ Menzies Institute for Medical Research, University of Tasmania, Hobart, TAS, Australia

**Keywords:** osteoarthritis, bioinformatics analysis, enrichment analysis, PPI network, microarray

## Abstract

The present study was performed to explore the underlying molecular mechanisms and screen hub genes of osteoarthritis (OA) *via* bioinformatics analysis. In total, twenty-five OA synovial tissue samples and 25 normal synovial tissue samples were derived from three datasets, namely, GSE55457, GSE55235, and GSE1919, and were used to identify the differentially expressed genes (DEGs) of OA by R language. The Gene Ontology (GO) and Kyoto Encyclopedia of Genes and Genomes (KEGG) analysis of DEGs were conducted using the Database for Annotation, Visualization, and Integrated Discovery (DAVID). A Venn diagram was built to show the potential hub genes identified in all three datasets. The STRING database was used for constructing the protein–protein interaction (PPI) networks and submodules of DEGs. We identified 507 upregulated and 620 downregulated genes. Upregulated DEGs were significantly involved in immune response, MHC class II receptor activity, and presented in the extracellular region, while downregulated DEGs were mainly enriched in response to organic substances, extracellular region parts, and cadmium ion binding. Results of KEGG analysis indicated that the upregulated DEGs mainly existed in cell adhesion molecules (CAMs), while downregulated DEGs were significantly involved in the MAPK signaling pathway. A total of eighteen intersection genes were identified across the three datasets. These include Nell-1, ATF3, RhoB, STC1, and VEGFA. In addition, 10 hub genes including CXCL12, CXCL8, CCL20, and CCL4 were found in the PPI network and module construction. Identification of DEGs and hub genes associated with OA may be helpful for revealing the molecular mechanisms of OA and further promotes the development of relevant biomarkers and drug targets.

## Introduction

OA, characterized by the destruction of articular cartilage and bone tissue, is one of the chronic diseases that seriously affect the elderly (Hutton, 1989; Zhou et al., 2018a). OA mainly affects the hip joints and knee joints (Rousseau and Garnero, 2012; Miller et al., 2014). Patients commonly experience pain and stiffness in the affected joint. Symptoms often appear after exercise at the beginning and become more frequent with the disease progression over time (Zhou et al., 2018b). The symptoms usually persist for many years that substantially impact the patients’ morbidity and quality of life. According to the data from epidemiological studies, OA affects approximately 237 million people (GBD, 2016), and about 10% of men and 18% of women over the age of 60 are affected. Approximately 1.9 million people in Australia and up to 53 million people in the United States are affected by OA (Cisternas et al., 2016).

Microarray technology is a tool commonly used in research in the fields of genetics and oncology, with important value in clinical applications, ranging from target therapy to molecular classification and patient stratification to prediction of the prognosis (Mi et al., 2018; Zhang et al., 2019). In recent years, many studies exploring the gene expression in OA were carried out using microarray technology, and some key genes and biomarkers have been found (Lu et al., 2014; He et al., 2016a; Lin et al., 2018). However, there are some drawbacks of microarray technology. The comprehensive analysis with respect to the identification of multiple factors that contribute to the development of OA has proven to be a major challenge. Analysis that incorporates expression profiling using bioinformatics analysis may be helpful for solving this problem. Approaches focusing on identifying the genes changed in OA and biomarkers are also needed for early diagnosis. Changes of gene expression may occur before the clinical symptoms become evident as well as the transformation of proteins or abnormalities in biomechanics.

In this study, we aimed to identify the signaling pathways and biomarkers associated with OA using a bioinformatics analysis. We downloaded the gene expression microarray data from the GEO database to search for DEGs and the associated biological pathways in OA by bioinformatics analyses. Analysis of the biological functions and pathways unique to OA may provide some useful insights into the mechanisms of the disease pathogenesis.

## Methods

### Tissue Sample Collection and Grading

The synovial tissues were extracted from the same site (medial space of the knee joint) of normal joints of patients suffering fatal accidents including death due to car accident or amputation due to trauma and OA (Kellgren–Lawrence grade Ⅲ to Ⅳ) patients undergoing total knee arthroplasty among the three databases enrolled in this study. All the samples were dissected immediately after surgery. Then, the tissues were snap-frozen in liquid nitrogen and stored at −80°C (Ungethuem et al., 2010; Woetzel et al., 2014).

The samples were carefully prepared and microscopically examined to remove the majority of the surrounding fat tissue. All the synovial membranes were graded using the inflammation score by Krenn to ensure consistency and comparability (Randen et al., 1995; Krenn et al., 2002).

### Inclusion and Exclusion Criteria

We used the following keywords: osteoarthritis, synovial tissue, and no intervention of drug to conduct a system search in the GEO dataset (https://www.ncbi.nlm.nih.gov/gds/). Our inclusion and exclusion criteria were as follows.

Inclusion criteria: 1) use case–control research design; 2) get the synovial tissue from the knee joint of the OA patient; and 3) includes normal control synovial tissue and osteoarthritis synovial tissue.

Exclusion criteria: 1) non-case–control research design; 2) non-synovial tissue from *Homo sapiens*; and 3) no normal control synovial tissue or osteoarthritis synovial tissue.

In total, two investigators (JZ and TL) independently checked each dataset, and we obtained three gene profiles (GSE55457, GSE55235, and GSE1919).

### Gene Expression Microarray Data

A total of three gene profiles (GSE55457, GSE55235, and GSE1919) comparing DEGs of synovial tissues between OA patients and healthy controls were downloaded from the GEO database. GSE55457 and GSE55235 contained 10 OA tissues and 10 normal tissues, and GSE1919 contained 5 OA tissues and 5 normal tissues. In the three datasets, 25 OA tissues were compared with 25 normal tissues.

### Normalization of Datasets

We used limma software of the Affy Bioconductor R package to preprocess the raw data for gene expression (http://www.bioconductor.org/packages/release/bioc/html/limma.html). Then, we performed quantile normalization and correction of the background of samples from each expression profile using RMA (robust multi-array average) of the R software Affymetrix toolkit. After we obtained the gene expression matrix, we used the aggregation function to calculate the mean of the amount of gene expression (Wu et al., 2013).

### Data Preprocessing and Differentially Expressed Gene Screening

We downloaded the sequence matrix files of three datasets and converted the probe names of each sequence matrix into gene symbols based on Affy probe annotation files. If multiple probes correspond to the same gene symbol, the aggregation function in R was used to average the expression value of that particular gene. The original data were preprocessed by the Affy package of R software 3.6.1, which was described in our previous study (Zhou et al., 2019a). Statistically significant DEGs were defined with the threshold for significant differential expression set at *p* < 0.05 and absolute log2-fold change (log2-FC) ≥1.

### Analysis of Hierarchical Clustering

We used the pheatmap package of R software 3.6.1 to conduct bidirectional hierarchical clustering analysis. The DEGs with similar expression patterns were clustered based on Euclidean distances of expression values. The details of this process were described in our previous study (Li et al., 2012; Zhou et al., 2019a).

### Gene Ontology and Kyoto Encyclopedia of Genes and Genomes Analysis

The GO database is a large set of gene annotation terms that can be used for annotating genes. The KEGG knowledge database is commonly used for exploring and analyzing gene functions and links between genomic information and higher-order functional information. DAVID is an important online data synthesis tool that provides the foundation for successful high-throughput gene functional analysis. In this study, we used GO, KEGG, and DAVID databases to analyze the GO functions and KEGG pathways of DEGs, which were described in our previous study (Zhou et al., 2019a).

### Protein–Protein Interaction Network Construction

STRING (https://string-db.org/), an online service, was utilized to obtain the interactions between the proteins encoded by DEGs. After that, we imported the data produced by the STRING online database into Cytoscape software to obtain the PPI network.

### MCODE Analysis

The MCODE serves to detect tightly connected regions in a PPI network. In the present analysis, we chose the important modules in the PPI network built by MCODE. The standard settings are as follows: node score cutoff = 0.2, K-Core = 2, and degree cutoff = 2. Then, we calculated the MCODE score.

## Results

### Normalization of Three Datasets

**FIGURE 1:**
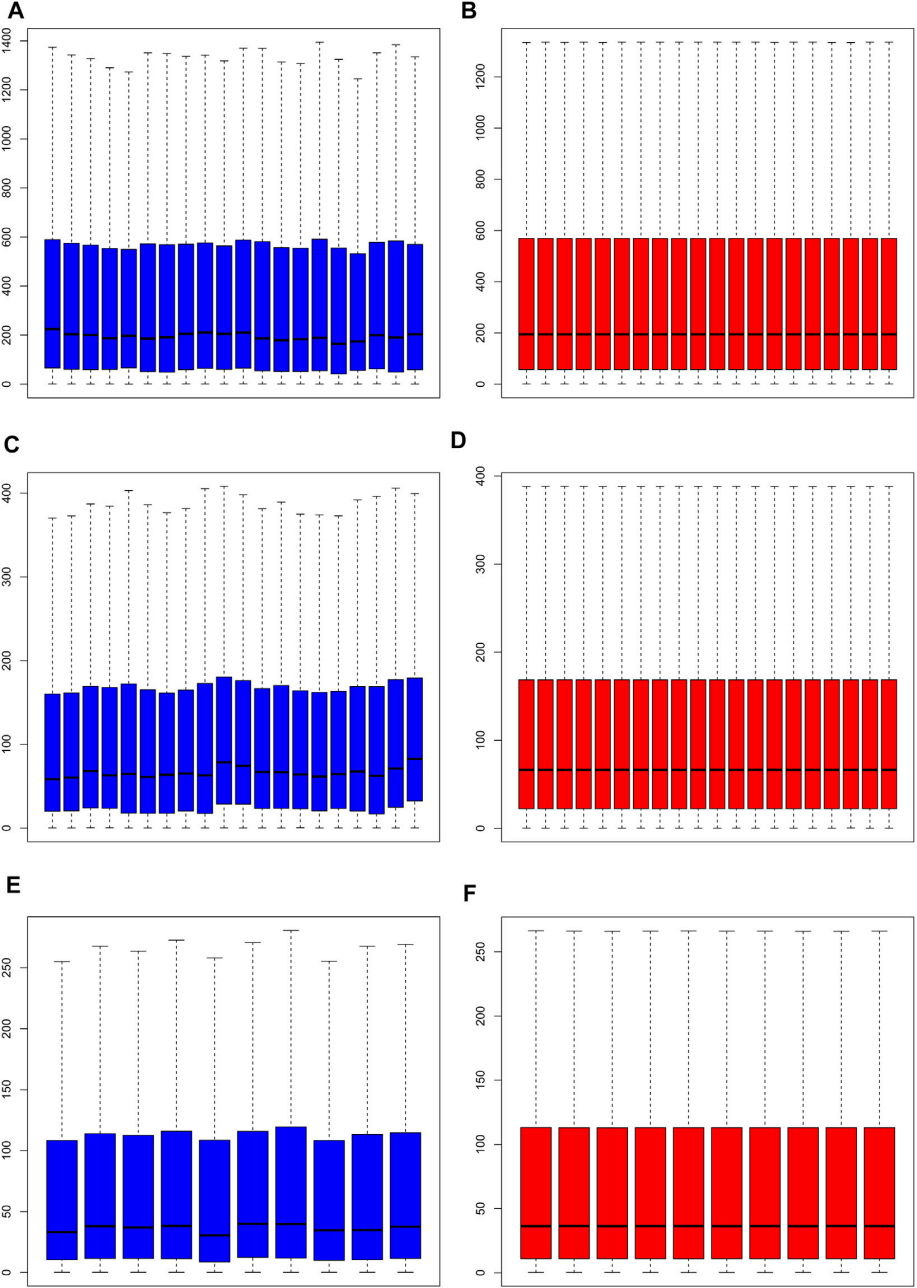
Normalization of samples. **(A, C, E)** Prior to normalization of total DEGs for GSE55457, GSE55235, and GSE1919; **(B, D, F)** following normalization of total DEGs for GSE55457, GSE55235, and GSE1919.

Normalization of the three datasets was performed *via* RMA of the R software Affymetrix toolkit. The results of normalization are shown in a boxplot in Figure 1. The normalization results of GSE55457 (Affymetrix Human Genome U133A Array), GSE55235 (Affymetrix Human Genome U133A Array), and GSE1919 (Affymetrix Human Genome U95A Array) are presented in Figures 1A–F, respectively. The black lines in Figures 1B,D,F are basically at the same level, presenting a high consistency.

### Differentially Expressed Genes in the Three Datasets

**FIGURE 2:**
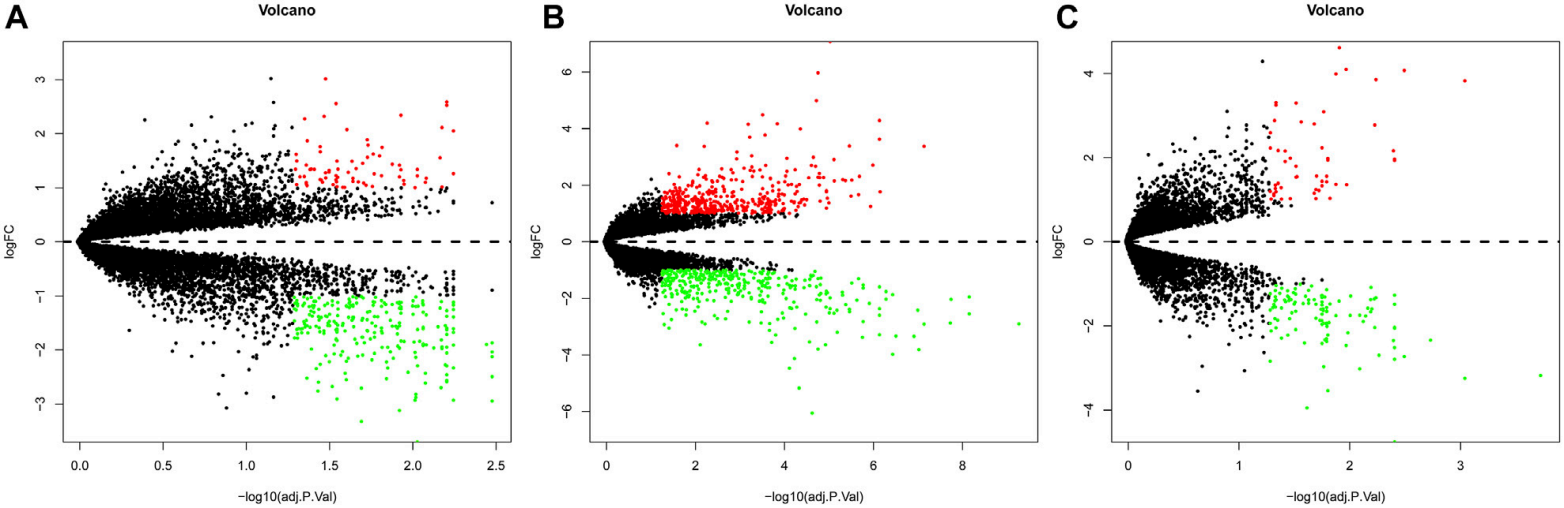
Volcano plot showing DEGs. **(A)** GSE55457, **(B)** GSE55235, and **(C)** GSE1919. Red dots: upregulated genes, green dots: downregulated genes, and gray dots: genes without change in expression.

**FIGURE 3:**
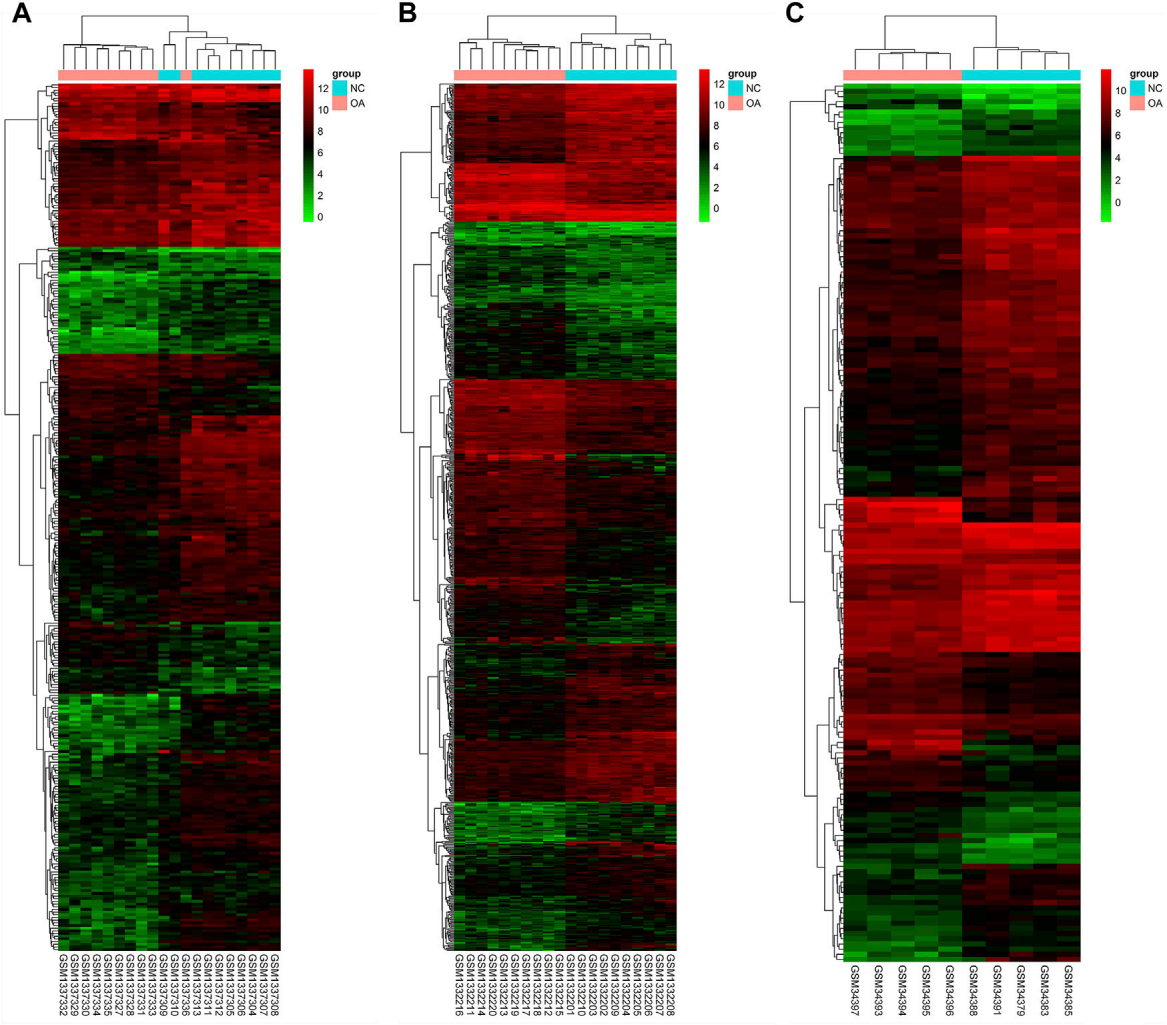
Heatmap showing DEGs. **(A)** GSE55457, **(B)** GSE55235, and **(C)** GSE1919. The expression values are log2 fold changes (>1 or < −1). Red color represents upregulation, and blue color represents downregulation.

Gene expression in the synovial membrane of OA patients was compared with that in the synovial membrane of healthy controls. We defined the statistically significant DEGs using the threshold as |log_2_FC| ≥1 and *p* < 0.05. A total of 1,127 DEGs were observed. Among them, 507 genes (GSE55457: 75, GSE55235: 425, and GSE1919: 54, 47 overlap genes) were upregulated and 620 genes (GSE55457: 249, GSE55235: 379, and GSE1919: 116, 124 overlap genes) were downregulated in OA patients compared with healthy controls. Expression volcano plots and heatmaps of all the upregulated and downregulated DEGs are shown in Figure 2 and Figure 3 (Figures 2A, 3A: GSE55457, Figures 2B, 3B: GSE55235, and Figures 2C, 3C: GSE1919).

### Analysis of Gene Ontology Enrichment

The DAVID database was used to analyze enriched GO terms and KEGG pathways of DEGs. All upregulated and downregulated genes were uploaded to DAVID. The results of the GO analysis showed that the upregulated genes were mainly enriched in immune response in the biological process (BP), extracellular region in the cell component (CC), and MHC class II receptor activity in the molecular function (MF). Downregulated genes were mainly involved in the response to organic substances in BP, extracellular region part in CC, and cadmium ion binding in MF (Table 1).

**TABLE 1:** Enriched GO terms of upregulated and downregulated expressed genes.

Ontology	ID	Description	Count	Adjusted *p*-value
Enriched GO terms of upregulated genes				
BP	GO:0006955	Immune response	56	1.50E-13
BP	GO:0006952	Defense response	42	4.62E-08
BP	GO:0002504	Antigen processing and presentation of peptide or polysaccharide antigen *via* MHC class II	10	1.27E-07
BP	GO:0007155	Cell adhesion	43	5.73E-07
BP	GO:0022610	Biological adhesion	43	5.93E-07
CC	GO:0005576	Extracellular region	108	1.03E-12
CC	GO:0044421	Extracellular region part	65	1.47E-11
CC	GO:0005578	Proteinaceous extracellular matrix	31	3.62E-09
CC	GO:0031012	Extracellular matrix	32	5.44E-09
CC	GO:0005887	Integral to plasma membrane	66	3.41E-08
MF	GO:0032395	MHC class II receptor activity	7	3.83E-06
MF	GO:0042287	MHC protein binding	7	2.21E-05
MF	GO:0030246	Carbohydrate binding	22	1.34E-04
MF	GO:0005201	Extracellular matrix structural constituent	10	2.16E-04
MF	GO:0001948	Glycoprotein binding	7	2.28E-04
Enriched GO terms of downregulated genes				
BP	GO:0010033	Response to organic substance	69	6.67E-19
BP	GO:0009725	Response to hormone stimulus	38	2.05E-11
BP	GO:0009719	Response to endogenous stimulus	39	9.20E-11
BP	GO:0048545	Response to steroid hormone stimulus	24	5.73E-09
BP	GO:0048511	Rhythmic process	19	2.19E-08
CC	GO:0044421	Extracellular region part	48	3.23E-06
CC	GO:0005615	Extracellular space	38	4.60E-06
CC	GO:0031981	Nuclear lumen	56	5.62E-04
CC	GO:0005667	Transcription factor complex	14	0.001961101
CC	GO:0005576	Extracellular region	69	0.002594958
MF	GO:0046870	Cadmium ion binding	7	9.50E-08
MF	GO:0003700	Transcription factor activity	53	6.72E-06
MF	GO:0043566	Structure-specific DNA binding	16	1.54E-05
MF	GO:0003690	Double-stranded DNA binding	12	9.17E-05
MF	GO:0005125	Cytokine activity	17	1.38E-04

### Analysis of the Kyoto Encyclopedia of Genes and Genomes Pathway

As shown in Table 2, the top 10 enriched KEGG pathways of upregulated DEGs were mainly involved in CAMs, viral myocarditis, asthma, antigen processing and presentation, intestinal immune network for IgA production, ECM–receptor interaction, allograft rejection, graft-versus-host disease, type I diabetes mellitus, and autoimmune thyroid disease. The top 10 enriched KEGG pathways of downregulated DEGs were mainly enriched in the MAPK signaling pathway, circadian rhythm, insulin signaling pathway, adipocytokine signaling pathway, Jak-STAT signaling pathway, cytokine–cytokine receptor interaction, toll-like receptor signaling pathway, neurotrophin signaling pathway, p53 signaling pathway, and prion diseases.

**TABLE 2:** Top 10 enriched KEGG pathways of upregulated and downregulated expressed genes.

Term	Pathway name	Count	Adjusted *p*-value
Top 10 enriched KEGG pathways of upregulated genes			
hsa04514	Cell adhesion molecules (CAMs)	20	4.73E-08
hsa05416	Viral myocarditis	13	2.86E-06
hsa05310	Asthma	9	2.98E-06
hsa04612	Antigen processing and presentation	13	1.53E-05
hsa04672	Intestinal immune network for IgA production	10	2.61E-05
hsa04512	ECM–receptor interaction	12	8.92E-05
hsa05330	Allograft rejection	8	1.46E-04
hsa05332	Graft-versus-host disease	8	2.48E-04
hsa04940	Type I diabetes mellitus	8	3.99E-04
hsa05320	Autoimmune thyroid disease	8	0.001332548
Top 10 enriched KEGG pathways of downregulated genes			
hsa04010	MAPK signaling pathway	25	2.42E-05
hsa04710	Circadian rhythm	5	8.99E-04
hsa04910	Insulin signaling pathway	14	0.001034299
hsa04920	Adipocytokine signaling pathway	9	0.002556227
hsa04630	Jak-STAT signaling pathway	12	0.023202188
hsa04060	Cytokine–cytokine receptor interaction	17	0.025604501
hsa04620	Toll-like receptor signaling pathway	9	0.028221432
hsa04722	Neurotrophin signaling pathway	10	0.033792237
hsa04115	p53 signaling pathway	7	0.034604644
hsa05020	Prion diseases	5	0.035607885

### Intersection Genes Among the Three Datasets

**FIGURE 4:**
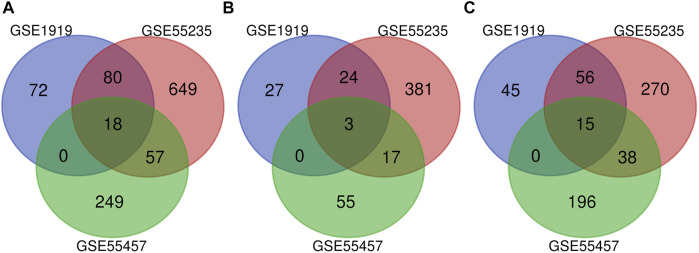
Venn diagram indicating the intersection genes among three datasets. **(A)** All DEGs, **(B)** upregulated DEGs, and **(C)** downregulated DEGs.

Cross-validation containing the intersection DEGs among the three datasets could help further examine hub genes of OA. A Venn diagram was used to show the intersection DEGs among the three datasets. According to the diagram, 18 potential hub genes exist in all the three datasets (Figure 4A). Among them, there were 3 upregulated DEGs (TRIL, NELL1, and SCRG1) and 15 downregulated DEGs (ATF3, SPRY1, RHOB, SLC2A3, DUSP5, NFIL3, STC1, CDKN1A, VEGFA, INHBB, KLF9, MAFF, TNFAIP3, SIK1, and GADD45B), which are shown in Figures 4B,C, respectively.

### Analysis of the Protein–Protein Interaction and Module

**FIGURE 5:**
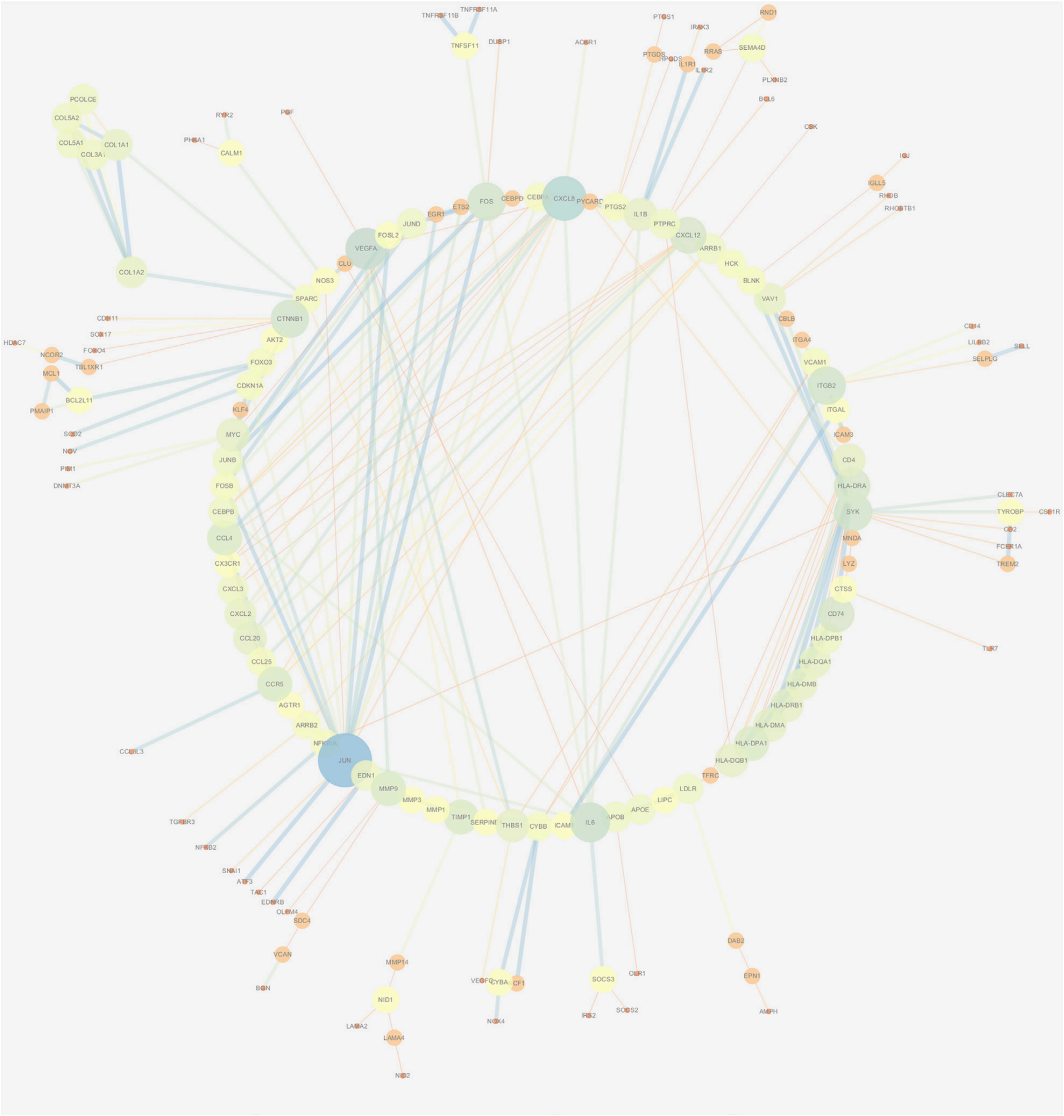
PPI network construction of DEGs. Low value of the combined score to small circle, circle with bright colors, and line with small sizes.

**FIGURE 6:**
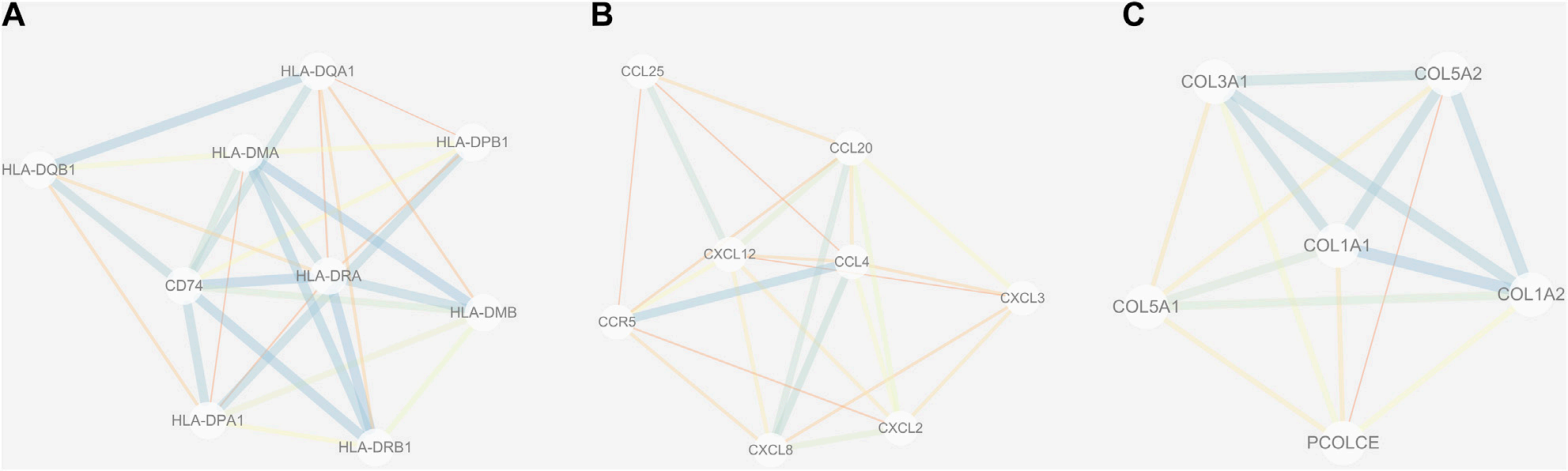
Three submodules **(A–C)** of the PPI network. Line with small sizes or bright colors: low combined score.

The PPI network of DEGs was constructed *via* the STRING (https://string-db.org/) database, and DEGs with a combined score ≥0.9 were subsequently visualized by Cytoscape (Figure 5). We set confidence score ≥0.4 as a cutoff value. MCODE analysis was conducted to show modules of the PPI network with a degree cutoff = 2, node score cutoff = 0.2, kcore = 2, and max depth = 100. A total of three significant modules were indicated with the parameter of a MCODE score ≥6. Module A (MCODE score = 7.25), Module B (MCODE score = 6.857), and Module C (MCODE score = 6) are shown in Figures 6A–C, respectively. Module A had 9 nodes and 29 edges involving 9 upregulated genes (HLA-DQA1, HLA-DQB1, HLA-DRB1, HLA-DRA, CD74, HLA-DPA1, HLA-DPB1, HLA-DMA, and HLA-DMB); Module B with 8 nodes and 24 edges involving 6 downregulated genes (CCL20, CXCL2, CXCL8, CCL25, CXCL3, and CCL4) and 2 upregulated genes (CCR5 and CXCL12); and Module C with 6 nodes and 15 edges involving 6 upregulated genes (COL5A2, COL5A1, COL3A1, COL1A1, COL1A2, and PCOLCE).

## Discussion

Osteoarthritis is a degenerative disease involving the entire joint including articular cartilage, subchondral bone, ligaments, joint capsule, and synovium (Zhou et al., 2019b; Hunter and Bierma-Zeinstra, 2019). In the present analysis, a total of 25 OA tissues were compared with 25 normal tissues in GSE55457, GSE55235, and GSE1919 to explore DEGs. A total of 507 upregulated genes and 620 downregulated genes were observed. However, the sample collection process and patient cohort of these three datasets may be largely different, which may contribute to substantial heterogeneity. Therefore, we performed the normalization with the same standard. Then, we plotted a Venn diagram to explore the intersection genes among these three datasets to identify the common DEGs. However, there is still some heterogeneity between datasets that cannot be ruled out and may affect the accuracy of the study findings. These include but are not limited to the differences in age, gender, and race between these three datasets.

The GO term analysis indicated that upregulated DEGs were mainly enriched in immune response in BP, extracellular region in CC, and MHC class II receptor activity in MF. Moreover, downregulated DEGs were mainly involved in response to organic substances in BP, extracellular region part in CC, and cadmium ion binding in MF. OA was considered a non-inflammatory disease in the past, but over recent years, it has been reported that low-grade inflammation with mild synovitis was linked to OA development (Berenbaum, 2013; Sokolove and Lepus, 2013). Existing evidence revealed that an early innate immune response plays an important role in the pathogenesis of OA. This process will lead to catalyzed degenerative changes, which ultimately result in an altered joint microenvironment (Kandahari et al., 2015).

In addition, the enriched KEGG pathways of upregulated DEGs were mainly involved in CAMs. Moreover, the enriched KEGG pathways of downregulated DEGs were mainly involved in the mitogen-activated protein kinase (MAPK) signaling pathway. According to previous reports, MAPK was a key signaling molecule in regulating cell proliferation and development. In addition, MAPK participates in morphogenesis and tissue patterning, which is important to chondrogenesis (Zhang et al., 2014).

To further identify important genes having a similar tendency in expression in those three datasets, cross-validation was conducted to explore DEGs using a Venn diagram. A total of 18 potential hub genes (3 upregulated and 15 downregulated) were identified in the three datasets. The chondroprotective effect of Nell-1 on OA has been evaluated by intra-articular injection. Nell-1 may be a potential method for the treatment of OA (Xiao et al., 2012). ATF3 is involved in the pathogenesis of OA by regulation of inflammatory cytokine expression in chondrocytes (Iezaki et al., 2016). RhoB was implicated in the activation of the functional phenotype of chondrocytes in OA (Gebhard et al., 2004). STC1 can suppress the proliferation of OA-FLS cells and promote apoptosis of OA-FLS cells (Wu et al., 2019). VEGFA is significant for chondrocyte survival (Zelzer et al., 2004). More studies are needed to understand the function of these genes.

A PPI network and three submodules with DEGs were constructed to explore the hub genes. CXCL12, CXCL2, CXCL8, CCL20, CCL4, HLA-DPA1, CD74, HLA-DRB1, HLA-DMA, and HLA-DRA were the top 10 hub genes. According to a previous study, CXCL12 may serve as an effective biomarker for the severity of OA (He et al., 2016b). CXCL8 may aggravate the disease progression of OA (Yang et al., 2016). CCL4 and CCL20 were significantly associated with the severity of X-ray-defined OA (Zhao et al., 2015; Lüderitz et al., 2018). HLA-DRB1 haplotypes were more frequently identified markers in OA (Rovetta et al., 2006).

Three modules (modules A, B, and C) were constructed to show the modules of the PPI network we obtained. Module A was important, and nine upregulated genes including HLA-DQA1, HLA-DQB1, HLA-DRB1, HLA-DRA, CD74, HLA-DPA1, HLA-DPB1, HLA-DMA, and HLA-DMB were involved in Module A. Previous reports indicated a linkage disequilibrium between HLA-DRB1 genes and genes included in the pathogenesis of OA (Moos et al., 2002). It would be interesting to explore the association between OA and other members from Module A, which may be helpful for understanding the pathogenesis of OA.

Module B was also significant. There were six downregulated genes (CCL20, CXCL2, CXCL8, CCL25, CXCL3, and CCL4) and two upregulated genes (CCR5 and CXCL12) enrolled in Module B. According to previous studies, several genes from Module B have been confirmed to be important for OA. The levels of CCL20 can reflect the OA severity (Guan et al., 2019). In addition, CXCL8 might be a novel therapeutic target for OA (Yang et al., 2016), and CCL25 may be a candidate for therapy approaches of cartilage repair (Lüderitz et al., 2018). Therefore, it is important to reveal the functional role of other genes from Module B, which may be interesting for discovering more therapeutic targets of OA.

Module C was also critical, and six upregulated genes including COL5A2, COL5A1, COL3A1, COL1A1, COL1A2, and PCOLCE were found in Module C. A previous study indicated that COL5A1 was involved in the OA synovium elevation of collagens and cross-linking enzymes (Tsezou, 2014). One previous study showed that COL3A1 may be a potential diagnostic biomarker for OA (Li et al., 2021). Another study demonstrated that the expression levels of COL1A1 and COL5A1 were significantly upregulated in OA (Zhu et al., 2020). COL1A2 was reported to be related to hip OA in the Newfoundland population (Snelgrove et al., 2005). These genes from Module C may serve as diagnostic or therapeutic targets for OA, while more experimental research is needed to confirm this observation.

A previous study has reported that PDGFRB, IFNG, EGR1, FASLG, and H3F3B from GSE48556 may be the potential targets for OA diagnosis and treatment (Feng and Lian, 2015). Huang et al. (2018) used GSE82107 to find that several molecular mechanisms were implicated in the development and progression of synovitis in OA. Bioinformatics analysis has now been widely used for predicting potential pathogenic genes and proteins, which provides new perspectives for disease diagnosis, treatment, and exploration of the underlying pathological mechanisms. It is convenient and effective to analyze the pathogenesis of OA using the GEO dataset. However, the number of samples in a single dataset was small, and the finding from a single gene profile was limited. Integrated analysis of multiple gene profiles was advantageous, and the conclusions may be more representative. Therefore, we performed integrated analysis using three gene profiles in this study.

There were several limitations in our study. First, the design including patient matching, differences in ages, differences in preparations, and difficulties of statistical considerations was not well handled. Second, in the present study, we did not conduct independent validation assays including qPCR, biochemical assays, and histology. Therefore, further experimental studies with validation analyses are needed to confirm our findings. Third, we did not perform any validation analysis to test whether the findings could be extrapolated to another dataset. Four, no statistical analysis was performed to specifically relate control to OA samples across studies. Five, we did not find the information of included patients in the GEO database; hence, no baseline data of included patients were given in this study.

## Conclusion

In summary, our study provides a comprehensive bioinformatics analysis of DEGs by comparing OA and normal synovial tissues, while more research on improving diagnosis of OA by regulating DEGs is still needed. The results in the present study provided a new insight into the molecular mechanism of OA, which may be helpful for future studies to identify new diagnostic biomarkers in the treatment of OA.

## Data Availability

The raw data supporting the conclusion of this article will be made available by the authors, without undue reservation.

## Abbreviations

BP, biological process; CAMs, cell adhesion molecules; CC, cell component; DAVID, Database for Annotation, Visualization, and Integrated Discovery; DEGs, differentially expressed genes; GO, Gene Ontology; KEGG, Kyoto Encyclopedia of Genes and Genomes; MCODE, Molecular Complex Detection; MF, molecular function; OA, osteoarthritis; PPI, protein–protein interaction; STRING, Search Tool for the Retrieval of Interacting Genes.
